# Smartphone Use and Addiction among Pharmacy Students in Northern Thailand: A Cross-Sectional Study †

**DOI:** 10.3390/healthcare11091264

**Published:** 2023-04-28

**Authors:** Dujrudee Chinwong, Pattarapan Sukwuttichai, Natthachai Jaiwong, Chalermpong Saenjum, Nuntaporn Klinjun, Surarong Chinwong

**Affiliations:** 1 Department of Pharmaceutical Care, Faculty of Pharmacy, Chiang Mai University, Chiang Mai 50200, Thailand; dujrudee.c@cmu.ac.th (D.C.);; 2 Center of Excellence for Innovation in Analytical Science and Technology for Biodiversity-Based Economic and Society (I-ANALY-S-T_B.BES-CMU), Chiang Mai University, Chiang Mai 50200, Thailand; 3 Department of Pharmaceutical Sciences, Faculty of Pharmacy, Chiang Mai University, Chiang Mai 50200, Thailand; 4 Faculty of Nursing, Prince of Songkla University, Songkhla 90110, Thailand; 5 Community Pharmacy Foundation, Volunteer Pharmacist Provide Smoking Cessation and Health Promotion, and Thai Health Promotion Foundation, Bangkok 10110, Thailand

**Keywords:** smartphone addiction, duration use of smartphone, pharmacy students, Thailand

## Abstract

In Thailand and worldwide, smartphone addiction among university students is a growing concern. This study aims to investigate behaviors of smartphone use, the prevalence of smartphone addiction, the duration of smartphone use, and their associated factors among pharmacy students at a university in northern Thailand. This cross-sectional study was conducted using an online self-administered questionnaire to collect data from January to February 2021. Smartphone addiction was measured using the Smartphone Addiction Scale: Thai Short Version (SAS-SV-TH). Of 281 students (70% female, average age of 21.1 (2.0), year 1 to 5), 87% used smartphones and tablets. Their average time spent on a smartphone was 7.5 (±3.1) hours daily on weekdays and 8.1 (±3.1) on weekends. The top three reasons for using smartphones were social networking (92.9%), education (90.3%) and entertainment (89.6%). Health-related problems associated with smartphone use were insomnia (51.3%), anxiety (41.3%), headache (38.8%) and stress (38.4%). The prevalence of smartphone addiction was 49% (95% CI: 44–55%); the associated factor comprised time spent on smartphones (>5 h/day). The prevalence of spending more than five hours daily on smartphones was 75% (95% CI: 70–80%) during weekdays and 81% (95% CI: 77–86%) during weekends; associated factors for during weekdays included a monthly smartphone bill of more than 500 THB (adjusted odds ratio: 4.30 (95% CI: 2.00–9.24) and for senior students (adjusted OR: 3.31 (95% CI: 1.77–6.19). The results remained the same for the weekend. In short, the results show that half of the pharmacy students were addicted to their smartphone; time spent on smartphones (>5 h/day) was associated with addiction. Therefore, university students should be encouraged to adopt healthy habits for smartphone use (such as limiting screen time and maintaining good posture while using a smartphone or tablet) and to increase their awareness of health-related problems.

## 1. Introduction

Smartphones and tablets are widely used in Thailand for various purposes, including communication, entertainment, online shopping and social media. According to a report by Hootsuite and We Are Social, as of January 2021, the number of mobile connections in Thailand exceeded 90 million, which is equivalent to approximately 130% of the population [1]. This indicates that many people in Thailand own multiple mobile devices or use dual-SIM cards. Overall, smartphones and tablets play a crucial role in the daily lives of many Thais, serving as a convenient and essential tool for communication, entertainment, education and commerce. According to a report by Hootsuite and We Are Social, as of January 2021, Thais spend an average of 8 h 44 min daily using the internet on any device, including smartphones, tablets and computers, whereas the average daily time using mobile internet was approximately 5 h [1]. This indicates that smartphones likely account for a significant portion of the time spent on the internet. Notably, the amount of time people spend using smartphones can vary depending on factors such as age, occupation and personal preferences. 

The use of smartphones among university students is common and has become an integral part of their daily lives. While smartphones can be useful for various academic and social purposes, they can also be a source of distraction and addiction, leading to overuse. Research studies have found that university students spend an average of four to six hours daily on their smartphones, with a significant proportion of that time spent on nonacademic activities such as social media, gaming and entertainment [2,3]. Overuse of smartphones can negatively impact academic performance, sleep quality and mental health [3,4,5,6,7]. According to a study by Ching SM et.al., smartphone addiction is “mainly characterized by excessive or poorly controlled preoccupations, urges, or behaviors regarding smartphone use, to the extent that individuals neglect other areas of life” [8]. The Smartphone Addiction Scale: Thai Short Version (SAS-SV-TH) was used in this study to measure smartphone addiction [9]. 

Several studies have investigated the impact of smartphone overuse among university students. A study by Thomée et al. found that excessive use of mobile phones was associated with increased psychological distress, sleep disturbances and symptoms of depression among university students [10]. Similarly, another study by Samaha and Hawi found a significant positive correlation between smartphone addiction and anxiety, depression and stress symptoms among university students [11].

Given the widespread use of smartphones and tablets in Thailand, this study aims to investigate behaviors of smartphone use, the prevalence of smartphone addiction and associated factors, and the prevalence of long durations of smartphone use and associated factors among pharmacy students at a university in northern Thailand.

## 2. Materials and Methods 

### 2.1. Study Design and Participants

This cross-sectional study was conducted among pharmacy students in a university located in northern Thailand. Only those students who met the following criteria were included: in their first to fifth year of pharmacy studies, aged 18 years or older, using of a smartphone and/or tablet, agreeing to participate in the study and having Internet access. Participants in their sixth year or those not providing adequate or reliable information were excluded from the study. The year 6 students were excluded due to their participation in a year-long clerkship rotation, resulting in a different daily routine compared to the year 1 to 5 students primarily focused on their studies.

The study determined the required sample size using Taro Yamane’s formula, considering a population size of 600 and a margin of error of 5.0% at a significance level of 0.05 for a two-sided test. The pharmacy program totaled approximately 600 students with approximately 120 students in each of the first five years. Based on these parameters, the estimated sample size for the study was 240 participants.

### 2.2. Questionnaire Development and Data Collection

A self-administered questionnaire was developed to answer all the objectives covering smartphone addiction, smartphone behavior, factors associated with smartphone addiction, and duration of smartphone use. The literature on smartphone behaviors, addiction and related issues was examined, and interviews were conducted with pharmacy students to ensure its applicability and relevance to their situation. Three experts in the field were responsible for evaluating the content validity of the questions. They used an index called Item–Objective Congruence (IOC), ranging from −1 to +1, with +1 indicating congruence, 0 indicating doubt and −1 indicating incongruence, to determine whether each question or answer was appropriate and relevant to the study’s goals. Questions with an IOC score of at least 0.5 were retained, while those with an IOC score of less than 0.5 were either modified or removed after consulting with subject matter experts. Next, the questions were tested for clarity and ease of understanding with a group of 30 pharmacy students. The questionnaire’s reliability was assessed using Cronbach’s alpha test, and the resulting coefficient of 0.89 indicated that the questionnaire was appropriate for use.

Finally, the self-administered questionnaire comprised three parts: general information, habits related to the use of smartphones and tablets, and smartphone addiction (Thai version), and included a cover page. The cover page explained the study’s purpose, and the students were given the option to participate voluntarily by selecting the statement “I consent to participate in the study voluntarily” before accessing the questionnaire. Participants were assured that their responses would remain anonymous. Section 1 included information about the respondents including sex, age, academic year, grade point average, place of residence, income from parents (THB/month) and smartphone bill (THB/month). 

Section 2 included the behaviors of smartphone and tablet use, including (1) the type of device used (smartphone only or smartphone and tablet), (2) the duration of smartphone use during weekdays (≤5 or >5 h/day), (3) the duration of smartphone use during weekends (≤5 or >5 h/day), (4) the purpose of use (such as social networking applications (Facebook, Instagram), educational purposes (online study, reading), entertainment (YouTube), playing games, and reading for non-academic purposes), and (5) health-related problems associated with smartphone use (a student was asked “Have you ever experienced any symptoms related to the use of smartphones or tablets?” You may choose more than one answer from the following options: headaches, insomnia, depression, anxiety, and stress).

Section 3 was to measure smartphone addiction and the details are listed under measured variables. 

The data collection from pharmacy students was conducted using an online questionnaire created through Google Forms. The representative of students each year was provided with a link to the finalized questionnaire. The respondents had the option to complete the questionnaire voluntarily and were free to discontinue at any point if they felt uncomfortable or hesitant to respond. The online questionnaire was open for two months, specifically from January to February 2021. Once the data was gathered, the researcher carefully reviewed each response to ensure that no duplicates existed.

### 2.3. Measured Variables 

Smartphone addiction was measured based on a tool called, “the Smartphone Addiction Scale: Thai Short Version (SAS-SV-TH) [9]. It contains ten items with Likert scale ratings of 1 to 6 (strongly disagree to strongly agree). The ten items were listed as follows: (1) “I didn’t do my planned work because of my smartphone.”, (2) “Due to using a smartphone, I find it difficult to focus in class, when working on assignments, or while performing other duties.”, (3) “I feel pain in my wrist or neck while using my smartphone.”, (4) “I feel restless every time without my smartphone.”, (5) “I would be frustrated if I wasn’t holding a smartphone in my hand.”, (6) “I always think about my smartphone even when I’m not using it.”, (7) “Despite the numerous negative effects using my smartphone has on my daily life, I can’t stop using it.”, (8) “I have to constantly check messages on my smartphone so I don’t miss a conversation with others on Twitter or Facebook.”, (9) “I tend to use my smartphone longer than intended.” and (10) “People around me tell me that I use my smartphone too much.”. The behavior of smartphone addiction is indicated by a score of greater than or equal to 33 points among women or greater than or equal to 31 points among men.

Duration of smartphone use was determined based on the literature review [12,13] and was divided in two groups: ≤5 h/day and >5 h/day. 

Other variables are listed in the section on questionnaire development. 

### 2.4. Statistical Analysis

Descriptive statistics, means and standard deviations (SD) for continuous variables, and frequencies and percentages for categorical data were used to display participant data. Fisher’s exact test for categorical data and the independent *t*-test for continuous variables were both used to compare the two groups. Using univariable and multivariable logistic regression, the factors associated with smartphone addiction were examined. The results were provided as a crude (for univariable analysis) and adjusted odds ratio (OR) (for multivariable analysis) with a 95% confidence interval (95% CI). Factors with *p*-value of less than 0.200 from the univariable logistic regression were then entered into the multivariable analysis, and variables were deemed statistically significant when their *p*-value was less than 0.05. Data analysis was performed using STATA Software, Version 14.0 (College Station, TX, USA).

### 2.5. Ethics Consideration

This study protocol was approved by the Ethics Committee of the Faculty of Pharmacy, Chiang Mai University, Thailand based on the Declaration of Helsinki, ICH GCP (Certificate of Approval No. 003/2021/E). All participants were informed about the study protocol and consented to be a part of this study. 

## 3. Results

Of 281 students (70% female, average age of 21.1 (2.0), years 1 to 5), 87% used both smartphone and tablet. Their average time spent on smartphone was 7.5 (3.1) hours daily for weekdays and 8.1 (3.1) for weekends (Table 1). 

The top three reasons for using smartphones were social networking (92.9%), education (90.3%) and entertainment (89.6%). Health-related problems associated with smartphone use included insomnia (51.3%), anxiety (41.3%), headache (38.8%) and stress (38.4%) (Table 2). 

The prevalence of smartphone addiction among pharmacy students was 49% (95% CI: 44 to 55%); one associated factor was time spent on smartphones (>5 h daily; adjusted OR: 2.56 (95% CI: 1.39 to 4.73) (Table 3). The prevalence of spending more than five hours daily on smartphones was 75% (95% CI: 70 to 80%) during weekdays and 81% (95% CI: 77–86%) during weekends; associated factors for during weekdays included a monthly smartphone bill of more than 500 THB (adjusted odds ratio: 4.30 (95% CI: 2.00 to 9.24) and senior students (adjusted OR: 3.31 (95% CI: 1.77 to 6.19). The results remained the same for the weekend (Table 4 and Table 5).

## 4. Discussion

### 4.1. Behaviors of Smartphone Use

The study found that the majority of students, specifically 87%, used both smartphones and tablets for their daily activities, whereas only 13% exclusively used smartphones. On weekdays, the average smartphone use time was 7.5 h, increasing to 8.1 h on weekends. Additionally, three quarters of the students spent more than five hours daily on smartphones during weekdays, and four fifths spent the same amount on weekends. Among the top three reasons for using smartphones, social media networks such as Facebook and Instagram, education and entertainment platforms such as YouTube were the most prevalent. The study also showed that one half of the students reported using smartphones for gaming or reading unrelated materials. More than half of the participants (51.3%) reported experiencing health issues, such as insomnia. However, the causality of these health issues with excessive smartphone use remains uncertain and requires further investigation. 

This study found that around 90% of students use smartphones for educational purposes. This was particularly relevant during the COVID-19 pandemic, when many universities worldwide shifted to online learning to reduce transmission risks. In the faculty where this study was conducted, particularly during the COVID-19 pandemic, smartphones and tablets served as essential tools for students to remain connected, organized, and engaged in their online coursework. However, students must use these tools responsibly and minimize distractions to ensure they are fully benefiting from their online learning experience. Furthermore, the findings of the present study suggest that the majority of students use their smartphones for noneducational purposes, such as engaging in social entertainment activities like browsing YouTube and using social networking platforms. These results are consistent with related research studies [12,13,14]. 

The excessive use of smartphones can significantly impact an individual’s health [10,11]. The findings of this study reveal that health-related problems associated with smartphone use include insomnia (51.3%), anxiety (41.3%), headaches (38.8%) and stress (38.4%). Although some students reported experiencing depression due to excessive smartphone use, this was an uncommon issue. Notably, some students experienced more than one health-related problem; the causative relationship between excessive smartphone use and these health issues remains unclear and requires further study. However, this study employed a cross-sectional design and, as such, could not establish a causal relationship between smartphone addiction and health issues. Future research should employ a cohort study design to investigate the causal relationship between smartphone addiction and health problems. This study’s findings were consistent with a study conducted by Thomée et al., showing that excessive use of mobile phones was associated with an increased risk of psychological distress, sleep disturbances and symptoms of depression among university students [10]. Additionally, a study by Samaha and Hawi found a significant positive correlation between smartphone addiction and symptoms of anxiety, depression and stress among university students [11]. 

### 4.2. Prevalence of Smartphone Addiction and Associated Factors

Fifty percent of pharmacy students exhibited symptoms of smartphone addiction. This finding was consistent with a study conducted in 2019 among pharmacy students at a university in central Thailand, reporting a lower prevalence of 39% [15]. In both studies, smartphone addiction was measured using the SAS-SV. However, this study was conducted during the COVID-19 era, which may have contributed to a higher prevalence of smartphone addiction among students. Its prevalence varied significantly across different regions and populations. A study conducted on 224 medical students of a tertiary care teaching hospital in North India reported a prevalence of 33.3% among females and 46.2% among males [16]. Very recently, a meta-analysis of multinational observational studies found that the prevalence of smartphone addiction among Asian medical students was 41.93% (95% CI [36.24 to 47.72%]) [17]. Moreover, the COVID-19 pandemic has also contributed to an increase in smartphone addiction rates as people rely more on their devices for communication, work and entertainment [18,19,20]. A study conducted in 2020 among Italian children and adolescents reported more frequent smartphone use during the COVID-19 pandemic compared with the pre-epidemic period [21].

After conducting multivariable logistic regression analysis, the findings indicate that only extended use of smartphones (more than five hours daily) was significantly associated with smartphone addiction. Pharmacy students using their smartphones for more than five hours daily on weekdays and weekends showed higher odds of developing a smartphone addiction compared with those using their smartphones less frequently. This finding is consistent with related studies, such as the research conducted by Alotaibi MS et al. in 2022, which identified those using an average smartphone of six to ten hours and for over eleven hours per day had a higher risk of developing smartphone addiction than those using their phones for under five hours, at 2.26 and 6.98 times, respectively [12]. Similarly, a study among young adults by Sohn SY et al. in 2021 reported that using a smartphone for more than three hours a day increased the risk of developing smartphone addiction, with the risk increasing by 2.45 times for those using smartphones for more than five hours daily [13]. In another study by Cha SS et al. in 2018, the amount of time spent using a smartphone was linked to an increase in the score on the smartphone addiction scale, with adolescents who use their phones for more than five hours daily being at a higher risk of developing smartphone addiction [22]. However, due to the cross-sectional nature of this study design, a causal relationship could not be established between prolonged smartphone use and smartphone addiction. 

### 4.3. Prevalence of Long Durations of Smartphone Use, and Associated Factors

The study found that pharmacy students spend an average of approximately 7.5 h daily on weekdays and 8.1 h daily on weekends using screens including smartphones and tablets. This was consistent with the statistics for the average Thai population aged between 16 and 64, who use electronic devices to access the internet for an average of 8.7 h daily [1]. Among pharmacy students, 75% use their smartphones for more than five hours daily during weekdays, while that percentage increases to 81% during weekends. These findings align with those of a study by Alotaibi MS et al. (2022), which reported that the majority of respondents used their smartphones for more than five hours daily [12].

After conducting a multivariable analysis, the findings indicate that students paying more than 500 THB monthly were more likely to spend more time on their smartphones during weekdays and weekends. Approximately 40% of students surveyed paid more than 500 THB monthly on their smartphone bills. Notably, the internet and smartphones are used for educational purposes and for nonlearning activities. Thailand offers many attractive internet promotion packages, with the cost directly correlating to increased internet stability, data use and speed. This could explain why students, who pay more for internet services, tend to use their smartphones for lengthier periods both academically and nonacademically. 

Based on the pharmacy curriculum, assignments for students can be designed for individual work and group collaboration. Such assignments may require students to engage in discussions or collaborations with peers and academic staff. The students in this study frequently use online platforms to collaborate with peers and discuss homework assignments. They also use these platforms to communicate with their teachers. The most commonly used platforms among participants are Zoom and Microsoft Teams. As a result, students spend more time on their smartphones and tablets during weekdays and weekends. Notably, senior students (years 4 and 5) are more likely to spend additional hours on their smartphones than junior students (years 1 to 3). This could be attributed to the higher volume of assignments given to senior students, requiring both individual and group work and necessitating the use of smartphones for research and information gathering. 

### 4.4. Public Health Implications

It is important to encourage university students to adopt healthy habits for smartphone use. According to the research, it is advisable to use a device at eye level with both hands and to take regular breaks to change positions during use. It is also recommended to limit the screen time spent using a smartphone in order to lower the risk of developing musculoskeletal problems [23]. Stakeholders involved in addressing smartphone addiction among university students, such as the university, faculty members, parents and guardians, and student organizations, should promote health interventions for good habits for smartphone use. Furthermore, the health promotion interventions should aim to raise awareness about health-related problems associated with excessive smartphone use. It is crucial for students to understand the potential risks that come with overusing smartphones and to take necessary measures to reduce their dependency on these devices. 

### 4.5. Limitations of This Study

The study findings should be interpreted with the consideration of several limitations. Firstly, the self-reporting nature of the questionnaire may have influenced participants’ perspectives and recalled experiences, leading to potentially biased responses. Secondly, the generalizability of the study results may be restricted to students in pharmacy or medical sciences, and may differ from those in nonmedical sciences. Thirdly, the analyses did not adjust for multiple comparisons, hence the findings should be interpreted with caution. Future studies should be conducted to confirm the robustness of the results. Fourthly, this study did not differentiate between hours spent for academic purposes versus recreational purposes; future studies should consider making this distinction to better understand hours spent for schoolwork purposes vs. hours spent recreationally on the smartphone. Additionally, the study was conducted during the COVID-19 pandemic, which could have affected the results. Finally, the cross-sectional study design employed in this study could not establish a causal relationship between long duration of smartphone use and smartphone addiction nor the association between higher bills and extended smartphone use. Therefore, a cohort study design is essential to determine whether prolonged smartphone use causes smartphone addiction or whether smartphone addiction leads to health-related problems.

## 5. Conclusions

In conclusion, half of the pharmacy students were addicted to smartphones, as measured by the SAS-SV, Thai version. The study also revealed a significant correlation between addiction and smartphone use, as students who spent more than five hours daily on their devices were more prone to addiction. Moreover, students with higher monthly smartphone bills (over 500 THB) tended to spend more time on their devices than those who had lower bills. Lastly, senior students used their smartphones for longer periods compared with junior students.

## Figures and Tables

**Table 1:** Characteristics of participants (*n* = 281).

Demographic Data	*n*	%
Sex		
Male	84	29.9
Female	197	70.1
Age (years)		
18–20	122	43.4
21–25	159	56.6
Mean (SD)	21.1 (2.0)	
Academic year		
1	44	15.7
2	77	27.4
3	31	11.0
4	76	27.1
5	53	18.9
Grade point average (GPA), *n* = 208		
2.01–2.99	95	45.7
3.00–4.00	113	54.3
Mean (SD)	2.99 (0.44)	
Place of residence		
House	33	11.7
Dormitory	248	88.3
Monthly incomes from parents (THB)		
≤3000	3	1.1
3001–5000	23	8.2
5001–7000	53	18.9
7001–9000	78	27.8
9001–12,000	100	35.6
>12,000	24	8.5
Smart phone or tablet monthly bill (THB)		
≤300	67	23.8
301–500	103	36.7
501–1000	92	32.7
1001–1500	17	6.1
1501–2000	1	0.4
>2000	1	0.4

**Table 2:** Behaviors of smartphone and tablet use among pharmacy students (*n* = 281).

Behavior	*n*	%
Electronic devices		
Smartphone only	36	12.8
Smartphone and tablet	245	87.2
Duration of smart phone and/or tablet use during weekdays (hours/day), mean (SD)		
≤5	69	24.6
>5	212	75.4
Mean (SD), min–max	7.5 (3.1), 1–19	
Duration of smart phone and/or tablet use during weekends (hours/day), mean (SD)		
≤5	53	18.9
>5	228	81.1
Mean (SD), min–max	8.1 (3.1), 2–19	
Purpose of smartphone and/or tablet use, *n*=269		
Using social network applications, e.g.,Facebook, Instagram	250	92.9
Education, e.g., online study, reading	243	90.3
Entertainment, e.g., Youtube	241	89.6
Playing games	150	55.8
Reading, not for education purpose	129	48.0
Health-related problems associated with smartphone use		
Headache	109	38.8
Insomnia	144	51.3
Depression	22	7.8
Anxiety	116	41.3
Stress	108	38.4

**Table 3:** Factors associated with smartphone addiction (*n* = 281).

Factor	NOT Addicted (*n* = 142)	Addicted (*n* = 139)	Crude OR (95% CI)	*p*-Value	Adjusted OR (95% CI)	*p*-Value
Sex						
Male	43 (30.3)	41 (29.5)	1.00			
Female	99 (69.7)	98 (70.5)	1.04 (0.62–1.73)	0.886		
Academic year						
1–3	74 (52.1)	78 (56.1)	1.00			
4–5	68 (47.9)	61 (43.9)	0.85 (0.53–1.36)	0.501		
Grade point average (GPA), *n* = 208						
2.01–2.99	50 (48.1)	45 (43.3)	1.00			
3.00–4.00	54 (51.9)	59 (56.7)	1.21 (0.70–2.09)	0.487		
Place of residence						
House	21 (14.8)	12 (8.6)	1.00		1.00	
Dormitory	121 (85.2)	127 (91.4)	1.84 (0.87–3.89)	0.113	1.76 (0.80–3.88)	0.158
Monthly income from parents (THB)						
≤9000	91 (64.1)	66 (47.5)	1.00		1.00	
>9000	51 (35.9)	73 (52.5)	1.97 (1.23–3.18)	0.005	1.36 (0.79–2.36)	0.267
Smart phone or tablet montly bill (THB)						
<500	98 (69.0)	72 (51.8)	1.00		1.00	
>501	44 (31.0)	67 (48.2)	2.07 (1.27–3.37)	0.003	1.41 (0.81–2.46)	0.227
Health problems						
Headache	50 (35.2)	59 (42.5)	1.36 (0.84–2.20)	0.214		
Insomnia	78 (54.9)	66 (47.5)	0.74 (0.46–1.19)	0.212		
Depression	11 (7.8)	11 (11.9)	1.02 (0.43–2.44)	0.958		
Anxiety	54 (38.0)	62 (44.6)	1.31 (0.82–2.11)	0.263		
Stress	48 (33.8)	60 (43.2)	1.49 (0.92–2.41)	0.107	1.50 (0.89–2.52)	0.127
Duration of smartphone use (hours/day)						
≤5	48 (33.8)	21 (15.1)	1.00		1.00	
>5	94 (66.2)	118 (84.9)	2.87 (1.61–5.12)	<0.001	2.56 (1.39–4.73)	0.003

**Table 4:** Factors associated with duration of smartphone use (≤5 h/day vs. >5 h/day) for weekdays (*n* = 281).

Factor	≤5 h/day (*n* = 69)	>5 h/day (*n* = 212)	Crude OR (95% CI)	*p*-Value	Adjusted OR (95% CI)	*p*-Value
Sex						
Male	24 (34.8)	60 (28.3)	1.00			
Female	45 (65.2)	152 (71.7)	1.35 (0.75–2.41)	0.308		
Academic year						
1–3	51 (73.9)	101 (47.6)	1.00		1.00	
4–5	18 (26.1)	111 (52.4)	3.11 (1.71–5.68)	<0.001	3.31 (1.77–6.19)	<0.001
Grade point average (GPA), *n* = 208						
2.01–2.99	23 (42.6)	72 (46.8)	1.00			
3.00–4.00	31 (57.4)	82 (53.3)	0.84 (0.45–1.58)	0.598		
Place of residence						
House	9 (13.0)	24 (11.3)	1.00			
Dormitory	60 (87.0)	188 (88.7)	1.18 (0.52–2.67)	0.700		
Monthly income from parents (THB)						
≤9000	49 (71.0)	108 (50.9)	1.00		1.00	
>9000	20 (29.0)	104 (49.1)	2.36 (1.31–4.24)	0.004	1.35 (0.69–2.64)	0.376
Smart phone or tablet monthly bill (THB)						
<500	58 (84.1)	112 (52.8)	1.00		1.00	
>501	11 (15.9)	100 (47.2)	4.71 (2.34–9.47)	<0.001	4.30 (2.00–9.24)	<0.001
Health problems						
Headache	22 (31.9)	87 (41.0)	1.49 (0.84–2.64)	0.177	1.27 (0.68–2.37)	0.447
Insomnia	34 (49.3)	110 (51.9)	1.11 (0.64–1.91)	0.706		
Depression	6 (8.7)	16 (7.6)	0.86 (0.32–2.28)	0.758		
Anxiety	30 (43.5)	86 (40.6)	0.89 (0.51–1.54)	0.670		
Stress	30 (43.5)	78 (36.8)	0.76 (0.44–1.31)	0.322		

**Table 5:** Factors associated with duration of smartphone use (≤5 h/day vs. >5 h/day) for weekends (*n* = 281).

Factor	≤5 h/day (*n* = 53)	>5 h/day (*n* = 228)	Crude OR (95% CI)	*p*-Value	Adjusted OR (95% CI)	*p*-Value
Sex						
Male	19 (35.9)	65 (28.5)	1.00			
Female	34 (64.1)	163 (71.5)	1.40 (0.75–2.63)	0.294		
Academic year						
1–3	36 (67.9)	116 (50.9)	1.00		1.00	
4–5	17 (32.1)	112 (49.1)	2.04 (1.09–3.85)	0.027	2.14 (1.10–4.14)	0.024
Grade point average (GPA), *n* = 208						
2.01–2.99	20 (45.5)	75 (45.7)	1.00			
3.00–4.00	24 (54.5)	89 (54.3)	0.99 (0.51–1.93)	0.974		
Place of residence						
House	7 (13.2)	26 (11.4)	1.00			
Dormitory	46 (86.8)	202 (88.6)	1.18 (0.48–2.89)	0.714		
Monthly income from parents (THB)						
≤9000	40 (75.5)	117 (51.3)	1.00		1.00	
>9000	13 (24.5)	111 (48.7)	2.92 (1.48–5.75)	0.002	1.68 (0.80–3.54)	0.174
Smart phone or tablet monthly bill (THB)						
<500	45 (84.9)	125 (54.8)	1.00		1.00	
>501	8 (15.1)	103 (45.2)	4.64 (2.09–10.27)	<0.001	3.56 (1.52–8.36)	0.004
Health problems						
Headache	15 (28.3)	94 (41.2)	1.78 (0.92–3.41)	0.084	1.48 (0.75–2.96)	0.256
Insomnia	29 (54.7)	115 (50.4)	0.84 (0.46–1.53)	0.575		
Depression	3 (5.7)	19 (8.3)	1.52 (0.43–5.32)	0.517		
Anxiety	17 (32.1)	99 (43.4)	1.63 (0.86–3.06)	0.133	1.44 (0.74–2.82)	0.287
Stress	22 (41.5)	86 (37.7)	0.85 (0.46–1.57)	0.610		

## Data Availability

The data presented in this study are available from the corresponding author on reasonable request.
